# Editorial: Natural feed additives in animal nutrition—Their potential as functional feed

**DOI:** 10.3389/fvets.2022.1062724

**Published:** 2022-11-09

**Authors:** Iveta Placha, Francesco Gai, Monika Pogány Simonová

**Affiliations:** ^1^Centre of Biosciences of the Slovak Academy of Sciences—Institute of Animal Physiology, Košice, Slovakia; ^2^National Research Council of Italy—Institute of Sciences of Food Production (CNR-ISPA), Grugliasco, Italy

**Keywords:** nutraceuticals, phytochemicals, probiotics, gut health, metabolism, oxidative stress, immunity

In recent years, natural products have become of great importance as growth-promoting agents to replace antibiotics. There is a social pressure on animal production industries to improve animal production performances, minimize economic losses, and ensure the safety of products for human consumption (1).

The potential negative consequences of the removal of antibiotics as growth promoters on gut health and growth performance have increased interest in finding alternatives to reduce the prevalence of bacterial infections and improve the quality of food products and animal performance by promoting gut health. In general, the term “gut health” represents the interaction between the microbiome, the intestinal wall barrier, and physiological and immune components, which allow different animals to cope with internal and external stressors (2).

Natural additives in animal feed are able to improve productivity and performance by enhancing digestibility and maintaining and stabilizing beneficial microflora in the gut; improving the quality of animal products can also positively influence the environment (3).

Natural feed additives such as prebiotics, beneficial microorganisms, bacteriocins, phytogenic compounds, and organic acids have potential as a research area for human or animal nutrition and health, and can satisfy the increasing consumer demand for natural substances. Since they are seen as novel valuable substances, their research is an ongoing discipline. Moreover, pharmacokinetics and the mechanism of action of phytogenic bioactive compounds are discussed and represent a novel approach (4).

Due to current restrictions on the use of antibiotics, particularly as feed additives, the objective of this Research Topic is to produce novel research results focused on the use of herbs, essential oils, prebiotics, probiotics, and postbiotics as alternative feed additives in animal nutrition.

This Research Topic aims to collect the latest research on the natural feed additives in animal nutrition. It covers a total of 21 articles (three reviews and 18 original researches) focused on three main topics: phytoadditives, probiotics/beneficial bacteria, and other natural additives as an alternative to antibiotic growth promoters with a beneficial impact on animal health. Most of the articles are related to monogastric animals, with more than half of the submissions focusing on poultry, while only two articles are related to ruminants.

Yang et al. examined in their study the effect of *in ovo* injection of *Astragalus* polysaccharide (APS) in broiler chickens and their results indicated that APS administered in this form did not affect hatchability but had the potential of promoting intestinal development and enhancing intestinal mucosal immunity in the early stage after hatching, which provides timely and effective protection for chickens.

Ghavipanje et al. determined the effect of pre- and post-partum supplementation of berberin (BBR) administrated in encapsulated form orally to goats. Taking all obtained results together, this study showed the potential of BBR as a novel strategy to mitigate oxidative stress and inflammation in dairy goats during the transition period.

To date, there have been few research studies on dietary supplementation with *Elephantopus scaber* L., used as a traditional herbal medicine in duck production. The authors of this study, Hu et al., provided basic knowledge on *E. scaber* addition to produce high-quality duck meat for human consumption. Based on their study, which contributes to a better understanding of the role of *E. scaber* as a feed additive in ducks, they recommended *E. scaber* as an effective supplement with beneficial impact on meat quality and intestinal development.

Phesatcha et al. investigated liquid-containing phytonutrients in dairy cows as dietary additives to reduce rumen protein degradation and confirmed their hypothesis that mangosteen peel liquid-protected soybean meal is able to improve milk yield and milk quality in lactating dairy cows.

Chen et al. showed that the natural polyphenol, chlorogenic acid (CGA), can attenuate oxidative stress-induced growth retardation and intestinal mucosa disruption by increasing antioxidant capacity and improving intestinal barrier integrity in weaned pigs. The authors explored this effect in pigs who were exposed to oxidative stress. They investigated the effect on growth performance, antioxidant activity, and the structure and functions of intestinal mucosa.

Darvishi et al. found that Licorice (*Glycyrrhiza glabra*) in the diet of rainbow trout fingerlings can increase their resistance to *Yersinis ruckeri* infection and effectively boost their immune system.

Microcine C7 is an antimicrobial peptide produced by *Escherichia coli*. Dai et al. evaluated its effect on growth performance, immune functions, intestinal barrier and cecal microbiota of broiler chickens. Obtained results indicated that Microcine C7 can be used as a promising alternative to traditional antibiotics. As an immunomodulator, it can regulate intestinal immune function and also exhibit antimicrobial activity against pathogen invasion. The enhancement of gut health improved the serum index and growth performance. To verify the specific mechanism responsible for these positive observations, future studies are needed.

Agradi et al. in their mini-review study evaluated the effects of Goji berries, the fruit of *Lycium barbarum*, supplementation in the diet of rabbits on reproductive and productive performances, immune system, metabolic homeostasis, and meat quality. Goji berries could determine health benefits for animals and consequently for consumers and have a role in optimizing production as well as in reducing the use of drugs. To find the correct dosage and period of its administration, further research is needed.

Recently, the application of soy lecithin has been dominant in poultry production, and only a few studies have reported the application of its hydroxylated form. Wu et al. described the positive effect of hydroxylated lecithin on growth performance, serum enzyme activity, hormone levels related to lipid metabolism, and the meat quality of Jiangan White goslings and recommended this form of lecithin as a safe and reliable additive in livestock production.

Based on current literature, recommendations for the addition of Paeoniae radix alba extract in raccoon dog diets are scarce. Wang et al. decided to obtain more information for its recommendation as a feed additive for raccoon dogs and they suggest Paeoniae radix alba extract in a concentration of 1–2 g/kg as optimal to achieve the optimal performance of Ussuri raccoon dogs.

To improve stability and bioavailability of essential oils and their compounds, the nanoemulsion as a novel strategy is recommended. Ibrahim et al. found that formulation of eugenol into the nanoemulsion form efficiently controlled its release in the gastrointestinal tract and thus boosted its bioavailability and broilers' response to avian pathogenic *Escherichia coli* (APEC) strains, especially O78, which causes colibacillosis with major economic losses. Obtained results could provide new insights for controlling colisepticemia induced by APEC.

The objective of the study by Luo et al. was to confirm the hypothesis that dietary supplementation of yucca powder may alleviate heat stress and improve growth performance in growing broilers. They confirmed that yucca could attenuate the heat stress and improve antioxidant status and feed intake probably by down-regulating the cholecystokinin in plasma and hypothalamus.

Chang et al. compared the effect of various oligosaccharides like isomaltooligosaccharide, raffinose oligosaccharide, and chitooligosaccharide on growth performance, immune and antioxidant functions, intestinal morphology, and microbiota in broiler chickens, and they concluded that oligosaccharides represent a very strong alternative to antibiotics.

The study by Danladi et al. demonstrates positive effects of postbiotics and paraprobiotics produced from *Lactiplantibacillus plantarum* supplementation in the broiler chicken diet on the intestinal microbiota. Administered additives increased concentration of beneficial microbes like *Firmicutes* while they decreased harmful microbes like *Proteobacteria***. **To achieve a healthier gut, the authors recommended postbiotics and paraprobiotics for modification of the microbiota in the intestine.

Numerous studies have investigated the potential of probiotics, prebiotics, synbiotics, and medicinal herbs as alternatives to antibiotics for the health management of aquaculture species. Wei et al. in their mini-review discuss the potential use of combinations of probiotics and medicinal herbs as prophylactic agents in aquaculture.

Ogbuewu et al. in their review indicated that *Bacillus* probiotics currently used in the broiler chicken industry are isolated from the gut, food, soil, and ponds. Probiotics may have achieved beneficial effects through various mechanisms of action. They are able to decrease pathogen proliferation in the gastrointestinal tract, increase production of organic acids leading to a decline in gut pH, improve production of antimicrobial compounds, improve oxidative stability and the immune system, and support the production and release of digestive enzymes. However, the potential of *Bacillus* probiotics in broiler chicken nutrition depends on a variety of factors such as *Bacillus* strain, species, dose level, and age of chicken. Further research is required to determine the optimum dose level before *Bacillus* probiotics could be considered as a substitute for antibiotics.

Chang et al. evaluated the potential of postbiotics originated from *Lactiplantibacillus plantarum* to modulate gut microbiota, mucin dynamics, and immune response in broiler chickens and recommended postbiotics as a good alternative to substitute antibiotic growth promoters in poultry.

The review by Damato et al. focuses on the characteristics and properties of clays as feed additives. It must be considered that clay minerals are not completely inert additives and can interfere with intestinal/ruminal metabolism with possible consequences on animal health. Further studies are needed, particularly on ruminants, to verify possible interferences of clays with rumen fermentations and metabolite uptake, which may affect animal metabolism and, possibly, milk characteristics. In particular, future studies should consider the effects of long-term administration or accumulation of clay minerals in organisms.

Azad et al. determined a beneficial replacement of corn-soybean meal with agricultural by-product cassava residue or fermented cassava residue, which could be a cost-effective dietary supplemental strategy for livestock production. These supplements increased the gut barrier function and beneficially altered gut microbiota composition.

Bai et al. confirmed that Dimethylglycine sodium salt (DMG-Na) can improve the redox status and relieve oxidative damage by scavenging the excessive generated free radicals in suckling piglets. They found that DMG- Na can act as a health-promoting additive in treating hepatic dysfunction in suckling piglets and is beneficial in improving their performance during the suckling period.

Guanidine acetic acid (GAA) is increasingly being considered as a nutritional growth promoter in monogastric animals. Whether or not such a response would exist in rapid-growing lambs is as yet unclear. Under a high-concentrate feed lotting pattern, the feed results obtained in the study of Li et al. indicated that daily gain presented a greater increase response to not only uncoated (GAA) but also coated (GAA) supplementation in young lambs with body weight from 13 to 30 kg, and such positive responses were more pronounced in oaten hay fed lambs in comparison with oaten hay plus wheat silage fed lambs.

In summary, the results of the above-mentioned research studies and reviews represent and summarize new relevant data on application of natural feed additives in animals. Despite all the existing research papers on this extremely important topic, the obtained information show that many aspects of bioavailability and beneficial doses of natural feed additives should be clarified. The presented and recommended future data on biological activity of natural substances in animal organisms could be suitable for pharmaceutical industries and the veterinary sectors.

## Author contributions

All authors listed have made a substantial, direct, and intellectual contribution to the work and approved it for publication.

## Conflict of interest

The authors declare that the research was conducted in the absence of any commercial or financial relationships that could be construed as a potential conflict of interest.

## Publisher's note

All claims expressed in this article are solely those of the authors and do not necessarily represent those of their affiliated organizations, or those of the publisher, the editors and the reviewers. Any product that may be evaluated in this article, or claim that may be made by its manufacturer, is not guaranteed or endorsed by the publisher.
